# Tone in politics is not systematically related to macro trends, ideology, or experience

**DOI:** 10.1038/s41598-023-49618-9

**Published:** 2024-02-08

**Authors:** Christian Pipal, Bert N. Bakker, Gijs Schumacher, Mariken A. C. G. van der Velden

**Affiliations:** 1 Department of Communication and Media Research, University of Zurich, Zurich, Switzerland; 2 Amsterdam School of Communication Research, University of Amsterdam, Amsterdam, The Netherlands; 3 Department of Political Science, University of Amsterdam, Amsterdam, The Netherlands; 4 Department of Communication Science, Vrije Universiteit Amsterdam, Amsterdam, The Netherlands

**Keywords:** Human behaviour, Anxiety

## Abstract

What explains the variation in tone in politics? Different literatures argue that changes in the tone of politicians reflect changes in the economy, general language, well-being, or ideology. So far, these claims have been empirically tested only in isolation, in single country studies, or with a small subset of indicators. We offer an overarching view by modelling the use of tone in European national parliaments in 7 countries across 30 years. Using a semi-supervised sentiment-topic model to measure polarity and arousal in legislative debates, we show in a preregistered multiverse analysis that the tone in legislative debates is not systematically related to previously claimed factors. We also replicate the absence of such systematic relationships using national leader speeches and parties’ election manifestos. There is also no universal trend towards more negativity or emotionality in political language. Overall, our results highlight the importance of multi-lingual and cross-country multiverse analyses for generalizing findings on emotions in politics.

## Introduction

Modern politics is overwhelmingly negative in tone^1^, and there is widespread belief that the tone of politicians is becoming increasingly more emotional and negative among academics (e.g^2–4^ and journalists (e.g.^5–8^). Politicians use their tone to get support for their policies (e.g.^9–12^). Yet, if it is correct that the tone of politicians is becoming more emotional and negative, then this is also worrying: a highly emotional and polarized political arena on the one hand encourages voters to vote with their gut instead of their mind, and on the other hand scares off voters disliking the tone^13^. Especially for marginalized groups the highly emotional and negative tone discourages them to enter politics amplifying unequal representation^14^. At a macro level, highly emotional and negative tone could prevent cooperation across political parties and ideological lines. But what explains this emotional and negative tone in politics?

Several grand theories contend to explain the rise in emotional and negative tone in the political arena. On the one hand, language, and therefore tone, is seen as fluctuating: for example, responsive to broader societal developments in general language use in society^15^ or as strategic responses to the changing electoral environment^16^ and audience^17^. On the other hand, tone is rather fixed, characterized by an ideological asymmetry between conservatives and liberals^18^. Research teams from different disciplines develop and test these theories in isolation with specific cases, unique operationalizations, and analysis strategies, in total producing scattered evidence (e.g.^4,16,19–23^).

We offer an overarching view using a multiverse approach that juxtaposes all data processing, variable operationalization, and modeling choices we deem reasonable^24^. This allows us to test multiple theories in multiple ways. In total, we estimate and present the results from 12,096 model specifications across seven European national parliaments and three decades, using legislative debate transcripts. These data speak directly to claims that the conduct of politics is becoming more negative and more emotional. This way we bridge disciplines, reconceptualize the dependent variable, and improve on research designs. Overall, we do not find support for a trend towards more negative or more emotional speeches in European parliaments. We are also not able to systematically replicate most previous findings on the determinants of tone in politics across countries. In addition, we also find no consistent evidence to support previous findings in our replication using election manifesto and leader speech data. This absence of evidence suggests that to explain the ups and downs in the tone of political language we require theories with a stronger role for context and agency.

In this paper, we are using the term tone. Terms such as sentiment, emotive rhetoric, and negativity have also been used to analyze the tone politician strike (e.g.^17,22,25^) in what we call political texts such as speeches, debates or election manifestos. In such texts politicians use emotional tone to engage with their audience. Following the core affect model^26^, tone differs in polarity (positive or negative) and arousal (emotional intensity). Tone can be used in both strategic or spontaneous utterances to convey a message. Thus, an important distinction here is that politicians who express emotional tone are not necessarily feeling emotional themselves.

What then explains the variation in tone? A finding that is systematically replicated is that (politicians of) government parties use more positive tone than (politicians of) opposition parties (e.g.^16,19–21,25^). In addition to this, various disciplines in the social sciences and humanities propose different arguments, to which we will turn now.

First, language itself changes. While common words are passed down through multiple generations for centuries, language evolves dynamically^27^ with some words getting in and out of fashion^28^. Also the meaning and concepts attached to words changes over time^29,30^. This is also the case for the emotions expressed in language. For instance, the language in American books, song lyrics, and newspapers has become more negative over the last decades^31,32^. At the same time, English language books became less emotional over time, while polarity fluctuates between periods^33^. Temporal changes are also observed in political language. For example, compared to previous decades, English-speaking politicians today use fewer words connected to analytical thinking^15^. Speeches of politicians also became less complex over time^34–37^. We, however, do not know if polarity and arousal in politics exhibit similar trends. Expecting that general language changes over time and political language following suit, we propose:

- **H1:** More positive general language use in society leads to more positive language of parliamentary debates.
- **H2:** More arousal in general language use in society lead to more arousal in the language of parliamentary debates.

Second, politicians have incentives for reelection, and therefore need to be perceived as responsive to (changing) citizen demands^38^. For example, a deep economic recession requires a response from politicians. Economic performance affects voting^39^, with government parties typically punished for poor economic performance, and opposition parties rewarded. Politicians, therefore, are incentivized to respond to changes in the economy. This may also be reflected in their tone: when the economy is underperforming politicians may be motivated to express the negative emotions felt by their constituents, when the economy is performing well they may wish to fuel a positive feeling in their constituents^16,19,40^. In total, we expect that the better the economy, the more positive the tone.

- **H3:** Increases in economic performance lead to more positive language of parliamentary debates.

We can also interpret the strategic perspective more broadly. Politicians have been found to be responsive to a public mood^41^. With mood political scientists refer to the public’s preference for a certain set of policies. For example, if citizens increasingly favor restrictive immigration policies, politicians follow or anticipate this and propose such policies^42^. Psychologist instead define mood as a long-lasting, persistent emotional state^43^. We define a public mood, then, as an aggregated persistent emotional state of citizens. Like the public mood of political science, politicians may be equally responsive to the public mood we define here. This means that politicians are incentivized, to some extent, to express the emotions their constituents feel. For example, in response to a national disaster, politicians may have to display sadness because this reflects the public mood. On the macro level, the public mood—how we define it—varies between countries and over time^44^. Importantly, this public mood does not necessarily become more positive as countries get richer^45^, suggesting that happiness includes more than economically motivated well-being. We hypothesize that these public moods should also be reflected in parliament, with politicians not only representing the policy preferences of their electorate, but also their emotional state.

- **H4:** Increases of subjective happiness in society lead to more positive language of parliamentary debates.

What these expectations have in common is that they are motivated by strategic considerations, and that politicians can change their tone. Psychology, however, also offers another perspective that argues that politicians should be much more consistent in tone in their political texts.

Particularly relevant is the negativity bias—the inclination to respond stronger to negative stimuli compared to positive ones. Conservatives, arguably, have stronger negativity biases than liberals do^46,47^. This is also reflected in the more negative tone conservatives use in political texts compared to liberals^18,48,49^. Negativity bias and ideology are rooted in personality, therefore, this a much more stable feature than any of the strategic conditions discussed. Psychologists consistently report correlations between personality and ideology. Personality describes a set of stable, long-term patterns of thinking, feeling and behaving. Personality traits such as extraversion, openness and conscientiousness systematically correlate with ideology, with for example low openness and high conscientiousness associated with conservatism. This is relevant because personality also correlates with tone^50–53^. For instance, neuroticism is linked to negative emotion experience^54,55^ and extraversion correlates positively with positive emotions^56,57^. Further, a study finds that both agreeableness and conscientiousness are negatively related to negative emotions^53^. Such patterns in the words we use (also think about use of pronouns, six-letter-words, words indicating vagueness) are relatively stable over time across a variety of contexts^52,58^. Personality is thus associated with preferences for tone and ideology. Because specific personality traits are more strongly associated with sociocultural conservatism than with economic conservatism^59–61^, we expect polarity to be most likely related to sociocultural conservatism:

- **H5.1:** Culturally conservative politicians speak more negatively than culturally liberal politicians.

There is, however, a growing body of research that more critically assesses the alleged ideological asymmetry between conservatives and liberals. For instance, recent studies did not find support for the link between negativity biases and political ideology in a series of cross-country psychophysiological experiments^62^ or for physiological correlates of ideology^63^. These results are in line with other recent work that failed to find evidence for a causal effect of personality on ideology^64–66^. In sum, the tone of conservative and liberal politicians might not differ after all. But even if there is a difference in negativity bias and tone, we don’t know if it is innate or can be strategically adapted to the goals of the politician. We thus also pose a contrasting hypothesis:

- **H5.2:** Cultural conservatism is not associated with the polarity in the language of parliamentary debates.

Within parties, politicians also need to build their personal brand and compete for resources such as public exposure. Especially new politicians are often limited in their public visibility and need to build a brand, a long-lasting associations between their name, their issue positions and communication strategy in a competitive market^67^. Emotions are typically an important part of a brand identity^68^. Political marketing views political parties and politicians from a brand perspective, assuming that voters make their electoral choices similar to how consumers make purchasing decisions^69^. To increase their visibility and recognition, politicians can attract attention by attacking their opponents^70^. In addition, this can benefit their reputation within their party for carrying out the “dirty” work of negative attacks. To increase their visibility, we expect less experienced politicians to speak more negatively.

- **H6.1:** More experienced legislators speak more positively in parliamentary debates.

Are they also more emotional than more experienced politicians? New politicians are also typically younger, and age generally correlates with a lower intensity of emotional experience^71^. Also, older people display emotions to a smaller extent and are more effective with regulating their emotions^72^. In addition, new and unexpected experiences can cause stronger emotional experiences^73^. We would thus expect that less experienced politicians are more emotional in their communication. We thus pose the following hypothesis:

- **H6.2:** More experienced legislators speak with less arousal in parliamentary debates.

It is difficult to compare and generalize previous findings (e.g.^4,16,19–23^ regarding the use of emotions in political speeches because they differ in samples of political texts, country selection, time selection, operationalization of variables and specification of statistical models (e.g. time-series models vs. multilevel models). We move beyond these limitations by adopting a multiverse approach^24^. This allows us to include all justifiable analytical choices regarding sample, operationalization and model specification and generate robust evidence.

We analyse the effects of these mechanisms on both the polarity and arousal of legislators’ tone in parliamentary debates using the ParlSpeech v2^74^ dataset. Parliamentary proceedings provide the perfect type of data because they are full records of political texts across long time spans, with comparable recording procedures between countries. The also speak directly to claims that the everyday conduct of politics is becoming more negative and emotional. We preregistered our study on OSF (https://osf.io/ur5xg/). Overall, we do not find evidence for any of our hypotheses. Instead, our multiverse analysis shows that the direction of the associations can go either way or are not statistically significant, depending on the specific model specification or country. To ensure our results are not driven by this specific type of data (parliamentary speeches), we replicated all analyses on two additional datasets that have been used in previous work on political communication: election manifestos^75^ and EU leader speeches^76^, also preregistered as a follow-up study (https://osf.io/v958t). Our conclusion remain the same.

## Results

**Figure 1:**
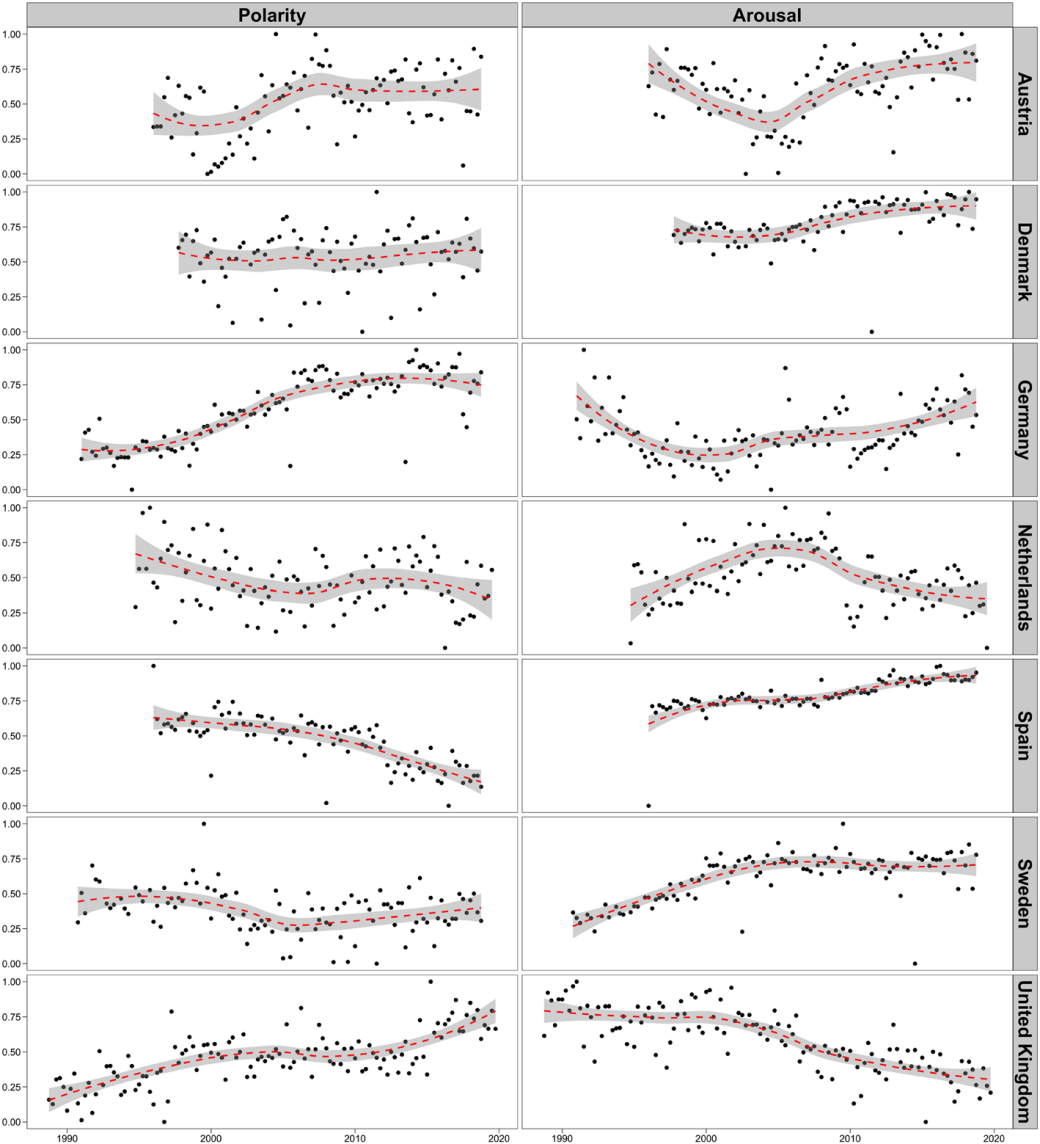
Speech polarity and arousal in European parliaments over time. *Note* The y-axis displays average polarity and arousal scores per quarter in each studied legislature (normalized by country). The red dashed line is a fitted regression line with 95% confidence intervals. Overall, the development of polarity and arousal over time differs between countries. Parliamentary debates became more positive in Austria, Germany, and the United Kingdom, but more negative in the Netherlands, and Spain. They also became more emotional in Denmark, Spain, and Sweden, but less emotional in the Netherlands and the United Kingdom.

To answer if tone in parliament indeed became increasingly negative and emotional over the last decades, Fig. 1 presents quarterly measures of speech polarity and arousal in the studied European parliaments. These measures have been normalized by country, thus negative (positive) values indicate quarterly scores below (above) the country mean. Overall, no uniform trends towards more negativity and emotionality are visible. Only in one country (Spain) parliamentary language became more negative, and a minority of European parliaments (Denmark, Spain, Sweden) show a trend towards more emotional language in parliament. This is in contrast to finding from the U.S., where language became more emotional^77^ and negative^4^. In sum, European parliaments show a heterogeneous picture when it comes to trends in the tone legislators strike.

Using a multiverse approach^24^, we specify up to 1296 multilevel models per country and dependent variable, each using different measurements of variables and model specifications. Each of these analytical choices is arbitrary in the sense that they are all equally justifiable. Table 2 in the “Methods” section gives an overview of all choices we considered. There we also explain each variable operationalization in detail. We measure polarity and arousal in the speeches of each individual legislator per quarter, using a semi-supervised sentiment-topic model^78–80^ seeded with validated multilingual Lexicoder sentiment dictionaries^81,82^. We explain this measure in detail in the “Methods” section (under Emotional polarity and Emotional arousal), and validate it in Appendix C. Our hypotheses, analytical strategy, and a replication using different data sources are preregistered (https://osf.io/ur5xg/). Deviations from these plans are explained in Appendix D. When interpreting effect sizes, it is important to keep in mind that even small changes in textual measures can indicate a substantive difference in the tone of a speech (see e.g.^16^).

**Figure 2:**
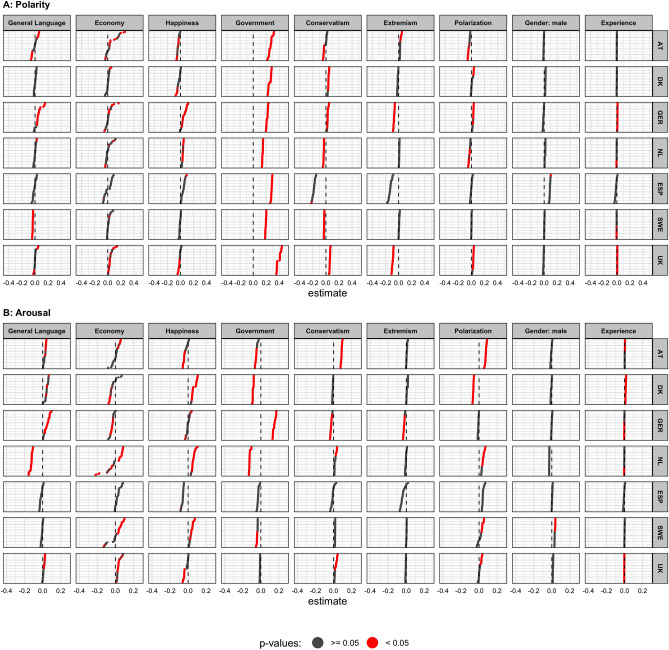
Specification curves of legislators’ tone across multiverse of models. *Note* Results from the multiverse of multilevel models explaining polarity and arousal in quarterly aggregated speeches of individual legislators (AT: n = 10,058, DK: n = 8526, GER: n = 33,326, NL: n = 10,357, ESP: n = 902, SWE: n = 18,641, UK: n = 57,141). Effects with $p <0.05$ are shown in red, effects with $p \ge 0.05$ are shown in grey. The dashed line indicates an effect of 0. All continuous variables have been standardized. To facilitate visual display, each specification curve depicts a random sample of 50 models. In panel (**A**), a total of 1296 models were estimated for each country (432 in panel **B**).

Figure 2 present the results from the multiverse analysis, and shows the estimated effects of 1,296 multilevel models per country (432 models for arousal). The dependent variable in panel A is the quarterly aggregated polarity of speeches given by an individual legislator. Panel B shows speech arousal. To illustrate, the top-right plot shows the specification curve of the experience variable on speech polarity in Austria. All dots of the specification curve are grey, thus each model estimate is insignificant with $p \ge 0.05$ using a two-sided test. In contrast, the top-left plot (the estimated effects of the general language polarity in Austria) includes statistically significant positive and negative effects shown in red. In this case, about 30% of the models result in a statistically significant effect of general language polarity on speech polarity in parliament. However, some of these effects are positive, and some are negative, giving an inconclusive picture.

### Preregistered analyses

We expected that with general language becoming more positive (H1) or more arousing (H2), parliamentary debates should follow in the same direction. Overall we do not find support for either hypothesis. Across all countries, we find conflicting results for the relationship between general language and tone in parliament. While in the majority of models the estimates are non-significant, the they also differ in direction between countries. Regarding polarity (H1) the results point into a positive direction in Germany, but towards a negative relationship in Sweden. The picture is similarly mixed for arousal (H2), with positive (Austria, Germany) and negative (Netherlands) estimates. Again, most results are not statistically significant.

We preregistered that increases in economic performance are positively associated with more positive language of parliamentary debates (H3). Again our results are inconclusive: For most countries we find both positive and negative, and mostly insignificant, estimates. The only exception is the United Kingdom, where our results show a positive relationship between economic performance and polarity across almost all multiverse specifications, replicating previous findings from this country (e.g.^20^).

Similarly to economic performance, we expected that increases of subjective happiness in society are associated with more positive language of parliamentary debates (H4). Across all models our results are inconclusive. While some models do produce statistically significant effects, they are positive in Germany and the Netherlands, but negative in Austria, Denmark, and the United Kingdom.

Turning to ideology, we had two competing hypotheses. H5.1 predicted ideological asymmetry (culturally conservative politicians speak more negatively than liberal politicians), while H5.2 predicted ideological symmetry (no difference between culturally conservative and liberal politicians). Overall, we do not find support for either hypothesis: Conservatism is associated with more negative tone in legislators’ speeches in Austria (half of the models), the Netherlands, and Sweden, but for some countries the effect is reversed (Denmark, Germany, United Kingdom). For Spain the results point towards a negative relationship, but the estimated effects are not statistically significant across the multiverse. While ideology and tone seem to be related, this relationship differs in direction between countries. We thus conclude that the effect of ideology on polarity is context-specific and cannot be generalized. In addition we looked at the effect of ideological extremism (not preregistered), and we expected more extreme politicians to speak more negatively. We do not find conclusive evidence for this either.

We formalize the interpretation of the effect of ideology on polarity across specifications using a bootstrapping procedure. We use bootstrap resampling to create a distribution of specification curves under the null hypothesis (no relationship between the independent and dependent variable), and test how inconsistent our obtained results are with the null hypothesis of no effect. We explain this procedure in detail in the “Methods” section (under Joint inference tests across multiverse specifications). Table 1 presents the results of these joint inference tests. Each line in the table presents the median effect of conservatism and extremism across the multiverse of specifications for each country, as well as the associated bootstrapped p-value. As already indicated visually in Fig. 2, there is no systematic effect of ideology (cultural conservatism or ideological extremity) on language polarity across countries. In all but one countries we do find a robust statistically significant association between ideology and polarity, but the direction of the association differs between countries. We thus conclude that we neither find support for the ideological asymmetry hypothesis H5.1 nor for the ideological symmetry hypothesis H5.2.

**Table 1:** Joint inference tests of the effects of ideology on polarity.

Independent variable	Country	Median effect across multiverse specifications	*p* value of bootstrap test
Conservatism	Austria	$-$ 0.02	0.43
	Denmark	0.04	≤ 0.01
	Spain	$-$ 0.37	≥ 0.99
	Germany	0.03	≤ 0.01
	Netherlands	$-$ 0.03	0.014
	Sweden	$-$ 0.04	≤ 0.01
	United Kingdom	0.05	≤ 0.01
Extremism	Austria	0.03	0.44
	Denmark	$-$ 0.02	≥ 0.99
	Spain	$-$ 0.04	≥ 0.99
	Germany	$-$ 0.07	≤ 0.01
	Netherlands	0.01	≥ 0.99
	Sweden	$-$ 0.00	≥ 0.99
	United Kingdom	$-$ 0.08	≤ 0.01

We also expected experienced politicians to speak more positively (H6.1) and with less arousal (H6.2). For both hypotheses our results provide conflicting evidence. In most countries the effects of experience on polarity are not statistically significant across the entire (Austria, Denmark, Spain) or majority (Sweden) of the multiverse. They are, however, consistently positive in Germany and the United Kingdom, but point towards a negative relationship in the Netherlands. Regarding arousal our results paint a similar conflicting picture.

### Exploratory analyses

In addition to our preregistered hypotheses we also explored additional correlates of tone in politics. Across countries and multiverse specifications, our single consistent finding is that legislators of government parties speak more positively compared to their colleagues from the opposition, replicating previous studies (e.g.^16,19–21,25^). We had no hypothesis about the relationship between ideology and arousal, and do not find evidence for ideological asymmetry when it comes to speech arousal. Unlike parliamentary debates in the U.S.^77^, there is no consistent relationship between speech arousal and ideology or ideological extremism. Previous studies found a relationship between political polarization and polarity in the United Kingdom^19^, with more positive language being used in times of low positional party polarization. We, however, find the opposite correlation across multiverse specifications in the United Kingdom, with similar results in Germany. In all other countries, our results are either not statistically significant or point in both positive and negative directions. Also, unlike previous studies^17,83,84^ we do not find a systematic relationship between gender and polarity or arousal. The only notably exception is Sweden were male legislators speak with more arousal compared to female legislators. We also explored interaction effects between the economy and government and between happiness and government in Appendix B. We do not find any systematic relationship here.

### Replication

Do our results generalize to other types of political text? To test this we replicated our analysis with two additional datasets: the Manifesto Project dataset^75^ and the EUSpeech v2 dataset^76^ using the 7 countries from the original analyses. These new analyses were preregistered after finishing the original analysis and follow the same procedure where possible. These results, presented in Appendix B.1 and B.2, do not change the general conclusion: there are no systematically positive or negative effects across countries and specifications.

## Discussion

Modern politics is overwhelmingly negative in tone^1^, and many believe that political language is increasingly negative and emotional (e.g.^2–8^). This has sparked interest in possible explanations of the ups and downs of emotions in politics (e.g.^16,19,20^). To systematically test several contending theories we conducted a multiverse analysis to explain the variation in the polarity and arousal of tone in 7 national parliaments. We find no evidence of a systematic effect of any of our theorized relationships. We do replicate the single, systematic finding that politicians from government parties use more positive tone than politicians from opposition parties.

Our multiverse approach produces findings across a large set of model and variable specifications. These results highlight that generalizations from existing theories, so far tested only in isolation and limited cases, are weak. They also illustrate that choosing a single specification could lead to biased conclusions. Other researchers might consider different analytical decisions, leading to a differently specified multiverse. Given the variation in the results, we believe it to be unlikely that an even larger multiverse leads to more systematic results. In sum, the multiverse approach produces a robust evaluation of the evidence.

In this paper we tell it like it is. Our results are statistically significant at times, but the direction of the effects varies between positive and negative across countries. Rather our suggestion is that we require theory with a stronger role for context and agency.

To illustrate, the ideological asymmetry claim stems from an assumed personality difference between liberals and conservatives. Personality is closely linked with linguistic habits, such as using particular emotion language. Yet, ideology does not explain all variation in personality. Therefore, personality may be linked to tone in a much richer way than ideology alone. For one, agreeableness is often linked with anger expressions but not with ideology. But also, personality may explain why two politicians would use entirely different tone in response to the same event. In sum, personality remains relevant, but rather as a way to theorize more complex individual-specific relationship with the polarity and arousal of tone.

Also, the hypothesised effects may be context-specific because not all mechanisms can work the same way in different political systems. For instance, national parliaments differ in the ability of individual members to speak, with high party control in most of them. It thus might be that the individual ability to strategically respond to changing environments is more pronounced in parliaments like the House of Commons where all members have an equal opportunity to speak. Different political systems also provide different electoral incentives to respond verbally. Further, it might also be the case that only specific time periods in the legislative cycle or types of debates allow for these mechanism to play out. The challenge here is that there are few countries we can compare, and many variables on which they differ.

While our study provides valuable insights using legislative debates, it is important to recognize the characteristics of this data source. Legislative debates might exhibit different trends in polarity and arousal compared to campaign advertising, news coverage, or social media communication. While legislative debates, like election manifestos or leader speeches, represent a highly formalized domain distinct from other platforms, they enable us to identify intentional decisions shaping the tone of politics. Therefore, our conclusions are limited to the platforms we have studied, and different patterns might emerge from campaign materials or other communication platforms. Future research would benefit from expanding our study to include these different platforms.

In addition, it’s worth exploring how political leaders might influence citizens’ emotional preferences. Drawing from research on the influence politics elites have on public preferences^85–87^ leaders might not just reflect public sentiment, but actively shape it. Understanding this potential feedback loop—where leaders’ tones impact public emotion—would enhance our understanding of political discourse dynamics.

Substantively, you may read a positive note in our conclusions. First, there is not a universal trend towards more negativity or more emotionality in political language. Rather, we observe ups and downs in polarity and arousal. Second, the suggestion that agency is more important than currently thought, proposes that politicians are not bound by some “iron cage” of emotionality, but that they themselves have a choice.

## Materials and methods

### Data

The data for our dependent variables is based on parliamentary speeches and come from the *ParlSpeech v2* dataset^74^. This dataset contains annotated full-text vectors of roughly 6.3 million plenary speeches in the key legislative chambers of Austria, the Czech Republic, Germany, Denmark, the Netherlands, New Zealand, Spain, Sweden, and the United Kingdom, covering periods between 21 and 32 years. The covered periods lie between 1987 and 2019. New Zealand is excluded from the analysis because of missing data in the independent variables. The Czech speeches were dropped because there is no Czech language stemmer in R. Additional data to measure independent variables come from the OECD Statistics Data^88^, the Manifesto Project dataset (MP)^75^, the ParlGov dataset^89^, and the Eurobarometer surveys

### Multiverse analysis

We follow a multiverse approach^24^ and display our results in the form of a specification curve^90^: Instead of only presenting one dataset and model resulting from our selection of variables, operationalizations, and modelling choices, we present the multiverse of datasets and models resulting from all choices we consider possible and reasonable. We then run all analyses on all of these datasets and present these results across all datasets and models. For each country we study, we use legislator level models (speeches aggregated by legislator and quarter) to test our hypotheses. We analyze each country individually, because formal and informal rules about who speaks in parliament differ between countries^91^. Table 2 lists the choice of variables and models we consider equally justifiable for the analysis. All options represent multiple ways the datasets and models can be constructed. The dependent variable is either polarity or arousal. In the remainder of this “Methods” section we provide additional details about these alternatives.

### Model specification

Our models deal with variation in our dependent and independent variables over time, within, and in-between legislators. To deal with this nested and time-dependent data structure, different ways of modelling have been employed in the literature on legislative speech making (e.g.^92,93^). The major difference compared to that literature is that our data deals with the content of legislative speeches, and not with the number of speeches given. We use multilevel models (quarterly measures clustered within legislators) using legislator random intercepts. To account for the repeated measurement of the dependent variables, we include models with a lagged dependent variable. In addition, following papers treating time as clusters on their own (e.g.^94^), we also estimate models with observations clustered within legislators and quarters, both with and without a lagged dependent variable. We also specify models with interaction effects between the government status of a legislator and economic performance/subjective happiness (see Table 2).

**Table 2:** Multiverse of model specifications.

Variables	Associated prereg. hypotheses or exploratory expectations
1. DV: Emotional polarity (P)/Emotional arousal (A)	
(a) P1: Net sentiment/(a) A1: Sum of sentiment probabilities	
(b) P2: Net sentiment weighted by arousal	
(c) P3: Empirical logit logit	
2. General language (L)	$\Rightarrow H1 \& H2$
(a) L1: Emotional polarity/arousal in movies	
(b) L2: Emotional polarity/arousal in books	
(c) L3: Mean of L1–L2	
3. Economic performance (EP)	$\Rightarrow H3$
(a) EP1: Economic growth rate	
(b) EP2: Recession (negative growth in two consecutive quarters)	
(c) EP3: Unemployment rate	
(d) EP4: Combined index of economic growth rate and unemployment rate	
4. Subjective happiness (H)	$\Rightarrow H4$
(a) H1: Subjective well-being (Eurobarometer)	
5. Conservatism (C)	$\Rightarrow H5.1 \& H5.2$
(a) C1: Cultural conservatism of legislator’s party	
6. Experience (EX)	$\Rightarrow H6.1 \& H6.2$
(a) EX1: Quarters in parliament since first elected	
7. Extremism (EXTR)	Extremism$$\uparrow$$$$\Rightarrow$$polarity$$\downarrow$$
(a) EXTR1: Cultural extremism of legislator’s party	
8. Polarization (POL)	$POL\uparrow \Rightarrow polarity\downarrow$
(a) POL1: Dalton’s polarization index	
9. Government status (G)	GOV:yes$\Rightarrow polarity\uparrow$
(a) G1: Member of government party	
10. Topic saliency (T)	None: control variable
(a) T1: Share of topic coverage of 5 meta topics	
11. Party size (P)	None: control variable
(a) P1: Seat share of legislator party	
12. Election (E)	None: control variable
(a) E1: Quarter of election	
(b) E2: Quarter preceding election	
(c) E3: Quarter preceding election + quarter of election	
13. Gender (G)	Difference, no direction
(a) G1: Gender of legislator (m/f)	
14. Models (M)	
(a) M1: Multilevel with legislator random intercepts	
(b) M2: M1 + lagged dependent variable	
(c) M3: Multilevel with legislator and quarter random intercepts	
(d) M4: M3 + lagged dependent variable	
15. Interactions (I)	
(a) I1: No interactions	
(b) I2: H x G	
(c) I3: EP x G	

### Joint inference tests across multiverse specifications

To simulate *p* values for inference tests across the multiverse of model specifications we use a bootstrapping-under-the-null procedure^95^. In contrast to a simpler permutation test this procedure does not assume randomly assigned independent variables, and is suitable for observational data^90^. We force the null-hypothesis of no effect on the data, and test how surprising our obtained results are given the null-hypothesis of no effect. Applying this approach to our multiverse analysis leads to the following steps:

1. We estimate all *K* specifications with the observed data. These result in *K* different point estimates: $\hat{b}_k$ with $k = 1 \ldots K$.
2. We generate *K* different dependent variables under the null, $y_k* = y_k - \hat{b}_k \times x_k$. Now every row of data has *x* values and *K* different $y*$ values.
3. We draw at random (with replacement) *N* rows from this dataset, using the same drawn rows of data for all K specifications.
4. We estimate the K specifications on the drawn data.
5. We repeat steps 3 and 4500 times.
6. For each bootstrapped sample we now have *K* estimates, one for each specification. We calculate how many of the resampled specification curves result in a significant effect at $\alpha = 0.05$. We then calculate the percentage of resamples that exhibit an equal or larger number of significant effects compared to the results from the observed data. This percentage is the p-value of the joint inference test.

### Emotional polarity

We use the polarity dimension of the core affect model^26^ to operationalize positive language use. We use a semi-supervised joint sentiment-topic model (JST)^78,79^ to measure polarity in the transcripts of parliamentary speeches. Compared to a plain application of sentiment dictionaries, this procedure improves the overall estimate of speech sentiment because it takes into account the context of a topic in which a word is used^80^, and we show a validation of the measure in appendix C. Importantly, JST models models take an emotion dictionary as input, and combine it with an unsupervised Latent Dirichlet Allocation (LDA) model^96^ that estimates the occurrence of topics in a text. To estimate these joint sentiment-topic model we use the *sentitopics* package for R. It follows the original implementation of JST^78^ and requires a sentiment dictionary as input. We use the Lexicoder Sentiment Dictionary^81^ and its machine-translated versions^82^, which have been extensively validated on political texts, including parliamentary speeches^82^. After feeding JST with these dictionaries, it estimates topic-specific sentiment and combines it into one overall document-level sentiment probability. For each text the model returns probabilities that the text is positive, negative, or neutral: If the model finds many words associated with positive topic-sentiment, the probability of the positive label increases. Vice versa, the probability of the negative label increases if the model finds many words associated with negative topic-sentiment. The literature operationalizes polarity in different ways. With our multiverse approach we can take into account these different specifications. The first option is to subtract the probability of negative sentiment from the probability of positive sentiment. This can be written as:

1 $$\begin{aligned} polarity = Pr(positive) - Pr(negative) \end{aligned}$$

    We also consider a transformation that weights the scores obtained from Eq. (1) by the arousal of a text (probability a text is positive or negative) in the following form:

2 $$\begin{aligned} polarity = \frac{Pr(positive) - Pr(negative)}{Pr(positive) + Pr(negative)} \end{aligned}$$

Finally, following other studies on parliamentary speech polarity^82^, we conceptualize polarity as the logged ratio of probabilities of positive to negative sentiment labels:

3 $$\begin{aligned} polarity = log\frac{Pr(positive) + 0.5}{Pr(negative) + 0.5} \end{aligned}$$

In all three cases, the polarity measure is bound between -1 (extremely negative) and 1 (extremely positive).

### Emotional arousal

Our second dependent variable reflects the intensity dimension of the Circumplex model. The measurement of this variable essentially follows the procedure described above. In fact we are using the same output from the JST model to measure arousal. To arrive at a continuous measure of arousal we sum the probabilities of the positive and negative labels. This can be written as:

4 $$\begin{aligned} arousal = Pr(positive) + Pr(negative)\ \end{aligned}$$

Since we are summing probabilities, this measure of arousal is bound between 0 and 1, where 0 indicates a text is entirely neutral (in this case the probability of the third, neutral category from the JST model would be 1), and 1 indicates a text is extremely emotional. This way, the difference between positive and negative probabilities can be small, while still containing high levels of arousal.

The preprocessing we apply to the speech data (text of speeches) includes removing speeches by the chair (who mainly cover procedural aspects), lower-casing, removal of non-alphabetic characters, removal of stopwords, and stemming. We also remove speaker-quarter observations with less than 50 words because they would make the measurement of the dependent variable unreliable. After this procedure, features that occur less than 10 times and in more than 95% of all documents within each legislature are removed to increase computational efficiency.

### General language

We use two data sources to measure the polarity and arousal of the general language used in a country: (1) the written words produced in each of the languages included in our dataset using the Google Books corpus, and (2) movie subtitles for each language using the opensubtitles.org corpus^97^. The polarity and arousal of the movie subtitles are measured using the Lexicoder dictionaries applied to the corpus, while for the Google Books corpus we used the Google Ngram Viewer, accessed via the ngramr R-package^98^, to look up the word shares of the words from the Lexicoder dictionaries in the respective language specific corpus and time period. We also use the (3) mean of these two measures. This allows us to investigate the combined effect of language change as well as differences between cultural institutions. Because the Google Books corpus is only available in German, English, and Spanish, the measurement of general language is limited to movie subtitles for Denmark, the Netherlands, and Sweden.

### Subjective happiness

Our measure of subjective happiness in society follows previous research on happiness and politics using self-reports of life satisfaction from large, representative samples (e.g.^99–102^). The variable is measured as the mean of all respondents per country to the life satisfaction question that has been asked in the Eurobarometer surveys since 1973. For that, the four ordinal answers of surveyed individuals are recoded into numeric values from 1 to 4. As our data source we use a dataset containing the combined Eurobarometer subjective happiness data from our studied time period^102^.

### Economic performance

To measure economic performance we use different indicators of economic prosperity. Economic growth is the percentage growth in real GDP expenditures from the OECD Statistics Data^88^. We consider the economy to be in a recession if growth is negative in two consecutive quarters. Also the unemployment rates come from the OECD Statistics Data. In addition, we create an index of economy prosperity by combining the growth and unemployment indicators. We include these different measures because different measures of objective well-being have been used in previous studies (e.g.^16,20^).

### Cultural conservatism and extremism

We measure cultural conservatism on the party level using a cultural liberal-conservative scale. Using the CMP dataset, we subtract attention to liberal issues in the dataset from the attention to the conservative issues. Liberal issues include pro-EU, pro-immigration, anti-national way of life, anti-traditional morality, pro-multiculturalism, anti-military, pro-internationalism, anti-imperialism, pro-peace, pro-environment, pro-culture, and support for under-privileged minority groups statements. Conservative issues include anti-EU, anti-immigration, pro-national way of life, pro-traditional morality, anti-multiculturalism, pro-military, anti-internationalism, pro-Freedom and Human Rights and pro-political authority, pro-law and order statements. Similar to previous work creating a composite scale from manifesto data (e.g.^37,103^), we use the log-transformed sums of attention to these individual issues. By squaring this score we arrive at the extremism measurement.

### Party system polarization

We measure party system polarization using the index by Dalton^104^. This index uses the left-right score of the CMP data, and calculates party system polarization as:

5 $$\begin{aligned} PI=\sqrt{\sum _i (party\_vote\_share_i)*([party\_LR\_score_i-party\_system\_average\_LR\_score]/5)^2} \end{aligned}$$

### Legislator experience

To measure the experience of a legislator in a given quarter, we count the number of quarters since the first time they entered parliament, independent of weather they served in parliament without interruptions.

### Government status

We control for the government status of the party a legislator belongs to. This is measured using the information on cabinet composition from the ParlGov dataset^89^, and equals 1 if a legislator’s party is in government during a quarter, and 0 otherwise.

### Topic saliency

We control for the selection of topics MPs speak about using the topic estimates of the joint sentiment topic model, which can be interpreted as the percentage a document is about a given topic. We aggregate these topics into 5 different thematic categories, and use these meta-topics for the further analyses. These topics are: (1) EU/European Integration, (2) Economy, (3) Security, (4) Healthcare, and (5) Immigration. The selection of these meta topics is based on an initial analysis of speeches from the UK ParlSpeech v1 data, where these meta-topics emerged as meta topics covering a large share of speeches.

### Party size

We also control for *party size*, measured as the seat share of the party a legislator belongs to using the ParlGov dataset^89^.

### Elections

We also control for possible effects of elections on speech polarity and arousal. We consider three ways to specify this variable and set a dummy variable to 1 for: (1) quarters preceding a general election, (2) quarters where an election takes place, and (3) quarters preceding a election and quarters where an election takes place.

### Supplementary Information

Supplementary Information.

## Data Availability

All data analyzed in this study are publicly available. Links to all data sources can be found in the preregistration document and the OSF project site: https://osf.io/ur5xg/.

## Code availability

All scripts used for the analyses presented in this study will be made available upon publication on the OSF project site: https://osf.io/ur5xg/.

## References

[1] Soroka SN (2014). Negativity in Democratic Politics: Causes and Consequences.

[2] Külz J, Spitz A, Abu-Akel A, Günnemann S, West R (2023). United States politicians’ tone became more negative with 2016 primary campaigns. Sci. Rep..

[3] Lengauer Günther, Esser Frank, Berganza Rosa (2012). Negativity in political news: A review of concepts, operationalizations and key findings. Journalism.

[4] Rhodes JH, Vayo AB (2019). The historical presidency: Fear and loathing in presidential candidate rhetoric, 1952–2016. Pres. Stud. Q..

[5] Caryl, C. The world is awash in the politics of fear. The Washington post (2018).

[6] Davies, W. How feelings took over the world. The Guardian (2018).

[7] Freeman, J. America descends into the politics of rage. The Atlantic (2018).

[8] Albert Arnold Gore (2007). The Assault on Reason: How the Politics of Blind Faith Subvert Wise Decision-Making.

[9] Erisen C, Villalobos JD (2014). Exploring the invocation of emotion in presidential speeches. Contemp. Politics.

[10] Ojala Maria, Cunsolo Ashlee, Ogunbode Charles A, Middleton Jacqueline (2021). Anxiety, worry, and grief in a time of environmental and climate crisis: A narrative review. Annu. Rev. Environ. Resour..

[11] Stapleton Carey E, Dawkins Ryan (2022). Catching my anger: How political elites create angrier citizens. Political Res. Q..

[12] Werlen, E., Imhof, C., & Bergamin, P. Emotions in the parliament: Lexical emotion analysis of parliamentarian speech transcriptions, in *SwissText* (2021).

[13] Lau RR, Pomper GM (2001). Effects of negative campaigning on turnout in U.S. senate elections, 1988–1998. J. Politics.

[14] Hobbs William, Lajevardi Nazita (2019). Effects of divisive political campaigns on the day-to-day segregation of Arab and Muslim Americans. Am. Political Sci. Rev..

[15] Jordan KN, Sterling J, Pennebaker JW, Boyd RL (2019). Examining long-term trends in politics and culture through language of political leaders and cultural institutions. Proc. Natl. Acad. Sci. U.S.A..

[16] Crabtree C, Golder M, Gschwend T, IndriĐason IH (2020). It is not only what you say, it is also how you say it: The strategic use of campaign sentiment. J. Politics.

[17] Osnabrügge M, Hobolt SB, Rodon T (2021). Playing to the gallery: Emotive rhetoric in parliaments. Am. Political Sci. Rev..

[18] Wojcik SP, Hovasapian A, Graham J, Motyl M, Ditto PH (2015). Conservatives report, but liberals display, greater happiness. Science.

[19] Kosmidis S, Hobolt SB, Molloy E, Whitefield S (2019). Party competition and emotive rhetoric. Comp. Pol. Stud..

[20] Rheault L, Beelen K, Cochrane C, Hirst G (2016). Measuring emotion in parliamentary debates with automated textual analysis. PLoS one.

[21] Schwalbach J (2021). Going in circles? The influence of the electoral cycle on the party behaviour in parliament. Eur. Political Sci. Rev..

[22] Valentim V, Widmann T (2021). Does radical-right success make the political debate more negative? Evidence from emotional rhetoric in German state parliaments. Political Behav..

[23] Widmann T (2021). How emotional are populists really? Factors explaining emotional appeals in the communication of political parties. Polit. Psychol..

[24] Steegen S, Tuerlinckx F, Gelman A, Vanpaemel W (2016). Increasing transparency through a multiverse analysis. Perspect. Psychol. Sci..

[25] Haselmayer Martin, Jenny Marcelo (2017). Sentiment analysis of political communication: Combining a dictionary approach with crowdcoding. Qual. Quant..

[26] Russell James A (1980). A circumplex model of affect. J. Pers. Soc. Psychol..

[27] Lieberman E, Michel JB, Jackson J, Tang T, Nowak MA (2007). Quantifying the evolutionary dynamics of language. Nature.

[28] Johnson E (1996). Lexical Change and Variation in the Southeastern United States, 1930–1990.

[29] Kozlowski, A. C., Taddy, M., & Evans, J. A. the geometry of culture: Analyzing meaning through word embeddings. *Am. Sociol. Rev.* 000312241987713 (2019).

[30] Rodman E (2019). A timely intervention: Tracking the changing meanings of political concepts with word vectors. Political Anal..

[31] Brand CO, Acerbi A, Mesoudi A (2019). Cultural evolution of emotional expression in 50 years of song lyrics. Evol. Hum. Sci..

[32] Iliev R, Hoover J, Dehghani M, Axelrod R (2016). Linguistic positivity in historical texts reflects dynamic environmental and psychological factors. Proc. Natl. Acad. Sci. U.S.A..

[33] Acerbi A, Lampos V, Garnett P, Bentley RA (2013). The expression of emotions in 20th century books. PLoS one.

[34] Lim ET (2011). The Anti-Intellectual Presidency: The Decline of Presidential Rhetoric from George Washington to George W. Bush.

[35] Spirling Arthur (2016). Democratization and linguistic complexity: The effect of franchise extension on parliamentary discourse, 1832–1915. J. Politics.

[36] Teten R (2003). Evolution of the modern rhetorical presidency: Presidential presentation and development of the state of the union address. Pres. Stud. Q..

[37] Schoonvelde Martijn, Brosius Anna, Schumacher Gijs, Bakker Bert N (2019). Liberals lecture, conservatives communicate: Analyzing complexity and ideology in 381,609 political speeches. PLoS one.

[38] Müller WC, Strøm K (1999). Policy, Office, or Votes?.

[39] Lewis-Beck MS (1986). Comparative economic voting: Britain, France, Germany, Italy. Am. J. Political Sci..

[40] Traber D, Schoonvelde M, Schumacher G (2019). Errors have been made, others will be blamed: Issue engagement and blame shifting in prime minister speeches during the economic crisis in Europe. Eur. J. Political Res..

[41] Stimson JA (1999). Public Opinion in America: Moods, Cycles, and Swings.

[42] Erikson RS, Mackuen MB, Stimson JA (2001). The Macro Polity.

[43] Ekman P, Davidson RJ (1994). The Nature of Emotion.

[44] Frey BS, Stutzer A (2002). What can economists learn from happiness research?. J. Econ. Lit..

[45] Easterlin RA, McVey LA, Switek M, Sawangfa O, Zweig JS (2010). The happiness - Income paradox revisited. Proc. National Acad. Sci. U.S.A..

[46] Hibbing JR, Smith KB, Alford JR (2014). Differences in negativity bias underlie variations in political ideology. Behav. Brain Sci..

[47] Tritt SM, Peterson JB, Page-Gould E, Inzlicht M (2016). Ideological reactivity: Political conservatism and brain responsivity to emotional and neutral stimuli. Emotion.

[48] Sylwester K, Purver M (2015). Twitter language use reflects psychological differences between democrats and republicans. PLoS one.

[49] Turetsky KM, Riddle TA (2018). Porous chambers, echoes of valence and stereotypes: A network analysis of online news coverage interconnectedness following a nationally polarizing race-related event. Soc. Psycholo. Personal. Sci..

[50] Hirsh JB, Peterson JB (2009). Personality and language use in self-narratives. J. Res. Pers..

[51] Lee CH, Kim K, Young SS, Chung CK (2007). The relations between personality and language use. J. General Psychol..

[52] Pennebaker James W, King Laura A (1999). Linguistic styles: Language use as an individual difference. J. Pers. Soc. Psychol..

[53] Yarkoni T (2010). Personality in 100,000 words: A large-scale analysis of personality and word use among bloggers. J. Res. Pers..

[54] Costa Paul T, McCrae Robert R (1980). Influence of extraversion and neuroticism on subjective well-being: Happy and unhappy people. J. Pers. Soc. Psychol..

[55] Larsen RJ, Ketelaar T (1991). Personality and susceptibility to positive and negative emotional states. J. Pers. Soc. Psychol..

[56] Lucas RE, Diener ED (2001). Understanding extraverts’ enjoyment of social situations: The importance of pleasantness. J. Personal. Soc. Psychol..

[57] Pavot W, Diener ED, Fujita F (1990). Extraversion and happiness. Personal. Individ. Differ..

[58] Words O, Selves O, James WP, Matthias RM, Kate GN (2003). Psychological aspects of natural language use. Annu. Rev. Psychol..

[59] Bakker BN (2017). Personality traits, income, and economic ideology. Polit. Psychol..

[60] Gerber AS, Huber GA, Doherty D, Dowling CM, Shang EH (2010). Personality and political attitudes: Relationships across issue domains and political contexts. Am. Political Sci. Rev..

[61] Malka A, Soto CJ, Inzlicht M, Lelkes Y (2014). Do needs for security and certainty predict cultural and economic conservatism? A cross-national analysis. J. Pers. Soc. Psychol..

[62] Fournier P, Soroka S, Nir Lilach (2020). Negativity Biases and political ideology: A comparative test across 17 countries. Am. Political Sci. Rev..

[63] Bakker BN, Schumacher G, Gothreau C, Arceneaux K (2020). Conservatives and liberals have similar physiological responses to threats. Nat. Hum. Behav..

[64] Ludeke S, Tagar MR, DeYoung CG (2016). Not as different as we want to be: Attitudinally consistent trait desirability leads to exaggerated associations between personality and sociopolitical attitudes: Not as different as we want to be. Polit. Psychol..

[65] Hatemi PK, Crabtree C, Smith KB (2019). Ideology justifies morality: Political beliefs predict moral foundations. Am. J. Political Sci..

[66] Hatemi PK, Verhulst B (2015). Political attitudes develop independently of personality traits. PLoS one.

[67] Ghodeswar BM (2008). Building brand identity in competitive markets: A conceptual model. J. Product Brand Manag..

[68] Whan Park C, Macinnis DJ (2018). Introduction to the special issue: Brand relationships, emotions, and the self. J. Assoc. Consum. Res..

[69] Reeves P, de Chernatony L, Carrigan M (2006). Building a political brand: Ideology or voter-driven strategy. J. Brand Manag..

[70] Dolezal M, Ennser-Jedenastik L, Müller WC (2017). Who will attack the competitors? How political parties resolve strategic and collective action dilemmas in negative campaigning. Party Politics.

[71] Diener ED, Sandvik ED, Larsen RJ (1985). Age and sex effects for emotional intensity. Dev. Psychol..

[72] Gross JJ, Carstensen LL, Pasupathi M, Tsai J, Skorpen CG, Hsu AYC (1997). Emotion and aging: Experience, expression, and control. Psychol. Aging.

[73] Frijda NH (1988). The laws of emotion. Am. Psychol..

[74] Rauh, C., & Schwalbach, J. The ParlSpeech V2 data set: Full-text corpora of 6.3 million parliamentary speeches in the key legislative chambers of nine representative democracies. Harvard Dataverse, v2 edition (2020).

[75] Volkens, A., Krause, W., Lehmann, P., Matthieß, T., Merz, N., Regel, S., & Weßels, B. The manifesto data collection. Manifesto project (MRG/CMP/MARPOR) (2019).

[76] Schumacher, G., Berk, N., Pipal, C., Kantorowicz, J., Schoonvelde, M., Traber, D., & Vries, E. D. EUSpeech V2 (2020).

[77] Gennaro G, Ash Elliott (2022). Emotion and reason in political language. Econ. J..

[78] Lin, C., & He, Y. Joint sentiment/topic model for sentiment analysis, in *International Conference on Information and Knowledge Management, Proceedings*, 375–384 (2009).

[79] Lin C, He Y, Everson R, Rüger S (2012). Weakly supervised joint sentiment-topic detection from text. IEEE Trans. Knowl. Data Eng..

[80] Pipal C, Schoonvelde M, Schumacher G (2021). Taking Context Seriously: Joint Estimation of Sentiment and Topics in Textual Data.

[81] Young L, Soroka S (2012). Affective news: The automated coding of sentiment in political texts. Polit. Commun..

[82] Proksch SO, Lowe W, Wäckerle J, Soroka S (2019). Multilingual sentiment analysis: A new approach to measuring conflict in legislative speeches. Legis. Stud. Q..

[83] Ennser-Jedenastik L, Dolezal M, Müller WC (2017). Gender differences in negative campaigning: The impact of party environments. Politics Gender.

[84] Hargrave L, Langengen T (2020). The gendered debate: Do men and women communicate differently in the house of commons?. Politics Gender.

[85] Canes-Wrone B (2010). Who Leads Whom?: Presidents, Policy, and the Public.

[86] Lenz GS (2012). Follow the Leader?: How Voters Respond to Politicians’ Policies and Performance.

[87] Soroka SN, Wlezien C (2010). Degrees of Democracy: Politics, Public Opinion, and Policy.

[88] OECD. OECD Stat: Quarterly national accounts (2019).

[89] Döring, H., & Manow, P. Parliaments and governments database (ParlGov): Information on parties, elections, and cabinets in modern democracies. *Infrastruct. Empir. Inf. Parties Elections Gov. Mod. Democr. Version* **12**(10) (2015).

[90] Simonsohn U, Simmons JP, Nelson LD (2020). Specification curve analysis. Nat. Hum. Behav..

[91] Proksch SO, Slapin JB (2014). The Politics of Parliamentary Debate: Parties.

[92] Bäck H, Debus M (2019). When do women speak? A comparative analysis of the role of gender in legislative debates. Political Stud..

[93] Bäck H, Baumann M, Debus M, Müller J (2019). The unequal distribution of speaking time in parliamentary-party groups. Legis. Stud. Q..

[94] Takens J, Kleinnijenhuis J, Van Hoof A, Van Atteveldt W (2015). Party leaders in the media and voting behavior: Priming rather than learning or projection. Polit. Commun..

[95] Flachaire E (1999). A better way to bootstrap pairs. Econ. Lett..

[96] Blei DM, Ng AY, Jordan MI (2003). Latent Dirichlet allocation. J. Mach. Learn. Res..

[97] Lison, P., & Tiedemann, J. OpenSubtitles2016: Extracting large parallel corpora from movie and TV subtitles, in *Proceedings of the 10th International Conference on Language Resources and Evaluation, LREC 2016*, 923–929 (2016).

[98] Carmody, S. ngramr: Retrieve and plot Google n-gram data, January (2023).

[99] Álvarez-Daz Á, Gonzlez L, Radcliff B (2010). The politics of happiness: On the political determinants of quality of life in the American states. J. Politics.

[100] Pacek A, Radcliff B (2008). Assessing the welfare state: The politics of happiness. Perspect. Politics.

[101] Tavits M (2008). Representation, corruption, and subjective well-being. Comp. Pol. Stud..

[102] Ward G (2019). Happiness and Voting: Evidence from four decades of elections in Europe. Am. J. Political Sci..

[103] Lowe W, Benoit K, Slava M, Laver M (2011). Scaling policy preferences from coded political texts. Legis. Stud. Q..

[104] Dalton RJ (2008). The quantity and the quality of party systems: Party system polarization, its measurement, and its consequences. Comp. Pol. Stud..

[105] Bernardi L, Bischof D, Wouters R (2020). The public, the protester, and the bill: Do legislative agendas respond to public opinion signals?. J. Eur. Public Policy.

[106] De Boef S, Keele L (2008). Taking time seriously. Am. J. Political Sc..

[107] Andreevskaia, A., & Bergler, S. Mining WordNet for fuzzy sentiment: Sentiment tag extraction from WordNet glosses, in *EACL 2006 - 11th Conference of the European Chapter of the Association for Computational Linguistics, Proceedings of the Conference* 209–216 (2006).

[108] Subasic P, Huettner A (2001). Affect analysis of text using fuzzy semantic typing. IEEE Trans. Fuzzy Syst..

[109] Van Atteveldt W, Van der Velden MA, Boukes M (2021). The validity of sentiment analysis: Comparing manual annotation, crowd-coding, dictionary approaches, and machine learning algorithms. Commun. Methods Measures.

[110] Widmann T, Wich M (2022). Creating and comparing dictionary, word embedding, and transformer-based models to measure discrete emotions in German political text. Political Anal..

