# Supplementary material for: Tone in politics is not systematically related to macro trends, ideology, or experience
Source: Sci Rep. 2024 Feb 8;14:3241. doi: 10.1038/s41598-023-49618-9 (PMC10853224; doi:10.1038/s41598-023-49618-9)
Supplement: Supplementary file 1 — Supplementary Information. [file 41598_2023_49618_MOESM1_ESM.pdf]

Appendix for: Emotional Appeals in Politics Are Not Systematically Related to Macro Trends, Ideology, or Experience

Christian Pipal[1], Bert N. Bakker[2], Gijs Schumacher[3], and Mariken A. C. G. van der Velden[4]

[1]Department of Communication and Media Research, University of Zurich

[2]Department of Political Science, University of Amsterdam

[3]Amsterdam School of Communication Research, University of Amsterdam

[4]Department of Communication Science, Vrije Universiteit Amsterdam

A Additional multiverse results

A.1 Government status interaction effects

Figure A1: Specification curves of legislator level interaction effects across multiverse of models

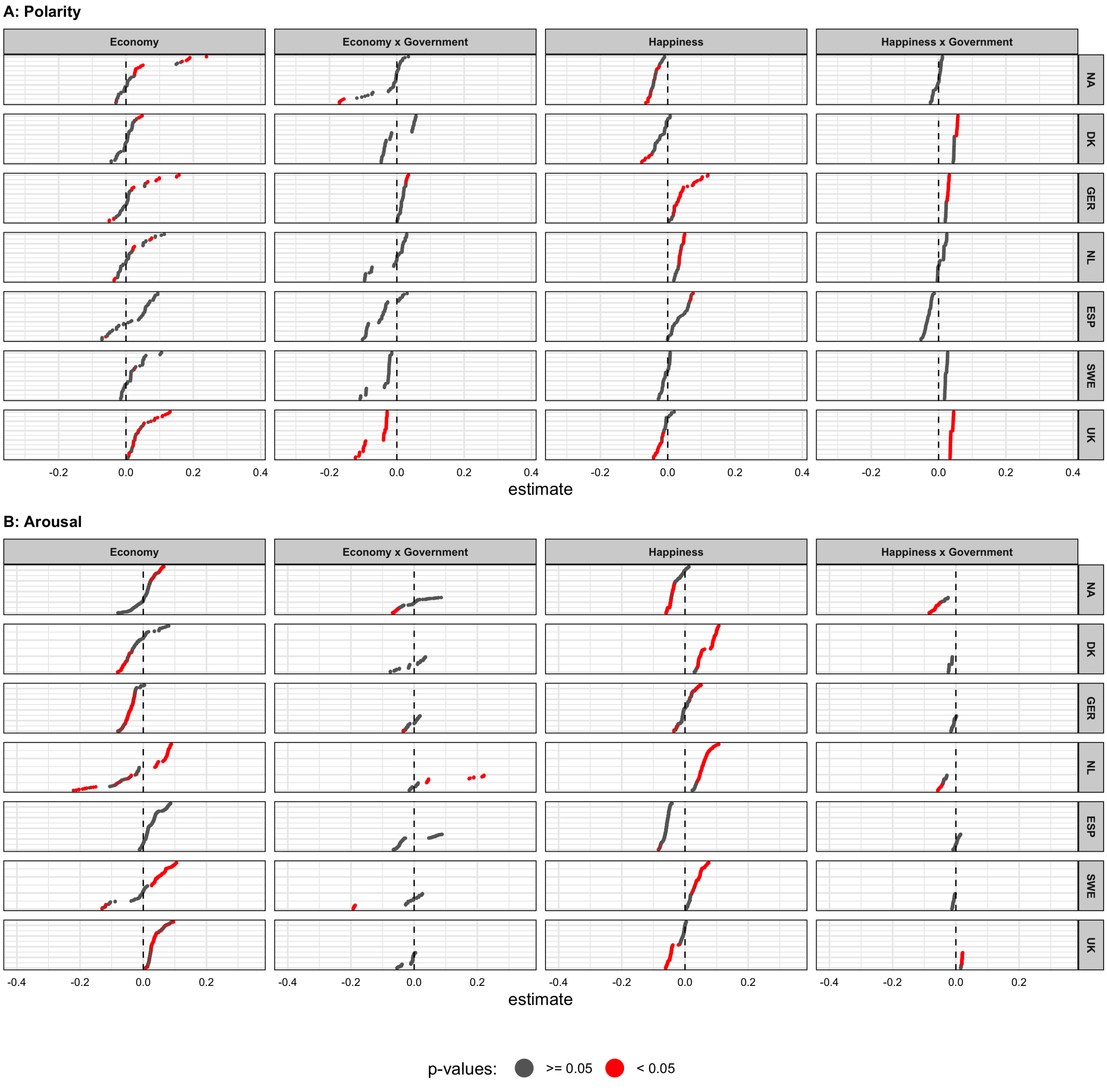

Note: Results from the multiverse of multilevel models explaining polarity and arousal in quarterly aggregated speeches of individual legislators. Effects with $p < .05$ are shown in red, effects with $p >= .05$ are shown in grey. The dashed line indicates an effect of 0. All continuous variables have been standardized.

A.2 Time series analysis (parliament level)

In the main text we modeled the relationship between our variables on the level of the individual legislator. Alternatively, we can also model it on the aggregated parliamentary level. In this case, parliament-quarter measures of tone are the dependent variable in a time series analysis. For this we use Error Correction Models (ECMs) which have been widely used i.a. in the analysis of parliament level legislative agendas (e.g. 1), and allow for an estimation of short- and long-run effects. Following the advice of De Boef and Keele (2) we use an unrestricted model, and specify the following form using a lag-structure of one time period:

$$\begin{aligned}
\Delta \mathrm{Y}_t = \beta_0 &+ \beta_1 \Delta \mathrm{Y}_{t-1} \\
&+ \beta_2 \Delta \text{General Language}_t && + \beta_3 \text{General Language}_{t-1} \\
&+ \beta_6 \Delta \text{Economic Performance}_t && + \beta_7 \text{Economic Performance}_{t-1} \\
&+ \beta_4 \Delta \text{Subjective Happiness}_t && + \beta_5 \text{Subjective Happiness}_{t-1} \\
&+ \beta_4 \Delta \text{Conservatism}_t && + \beta_5 \text{Conservatism}_{t-1} \qquad (1) \\
&+ \beta_4 \Delta \text{Extremism}_t && + \beta_5 \text{Extremism}_{t-1} \\
&+ \beta_4 \Delta \text{Polarization}_t && + \beta_5 \text{Polarization}_{t-1} \\
&+ \xi_t \\
&+ \epsilon_t
\end{aligned}$$

with the dependent variable being $\Delta \mathrm{Y}_t$ which denotes the change in polarity or arousal in parliament speeches between time t and $t-1$, β_0 the intercept, ξ a set of control variables equal to the analysis in the main text, and ϵ the error term.

Figure A2 presents the results from all parliament level multiverse analyses, and shows the estimated effects of 864 ECM models per country (288 models for arousal). The dependent variable in panel A is the quarterly measure of speech polarity. Panel B shows the same distribution of effects on speech arousal. The delta variables indicate the estimated effects and p-values of the differenced independent variables (the equilibrium term of the ECM), lagged variables indicate the estimated effects and p-values of the lagged independent variables (the transient term of the ECM).

The results of the time-series analysis is in line with results from using legislator-level models: None of our independent variables is associated with either polarity or arousal consistently across countries and model specifications.

Figure A2: Specification curves of parliament level tone across multiverse of models

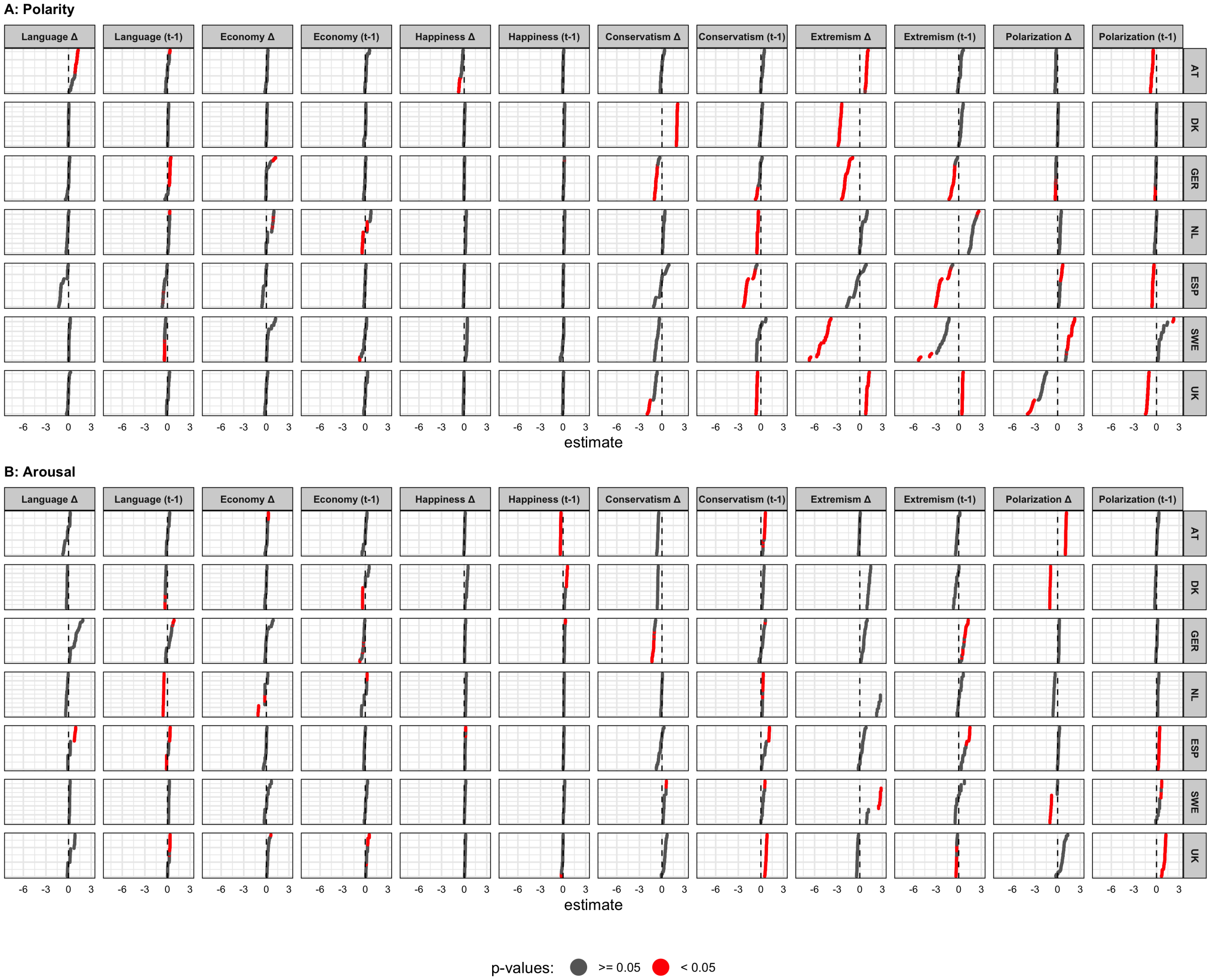

Note: Results from the multiverse of ECM time series models explaining polarity and arousal in quarterly aggregated parliamentary speeches (AT: n = 72, DK: n = 76, GER: n = 92, NL: n = 88, ESP: n = 83, SWE: n = 97, UK: n = 113). Effects with $p < .05$ are shown in red, effects with $p >= .05$ are shown in grey. The dashed line indicates an effect of 0. All continuous variables have been standardized. The number of models for Denmark, the Netherlands, and Spain is 66% lower because the data sources for one variable (General Language) are limited for these countries. The measures for conservatism and extremism have been averaged on the parliament level for this analysis.

B Replication with alternative data sources

B.1 Replication with election manifestos

Figure B1: Specification curves of tone in party manifestos across multiverse of models

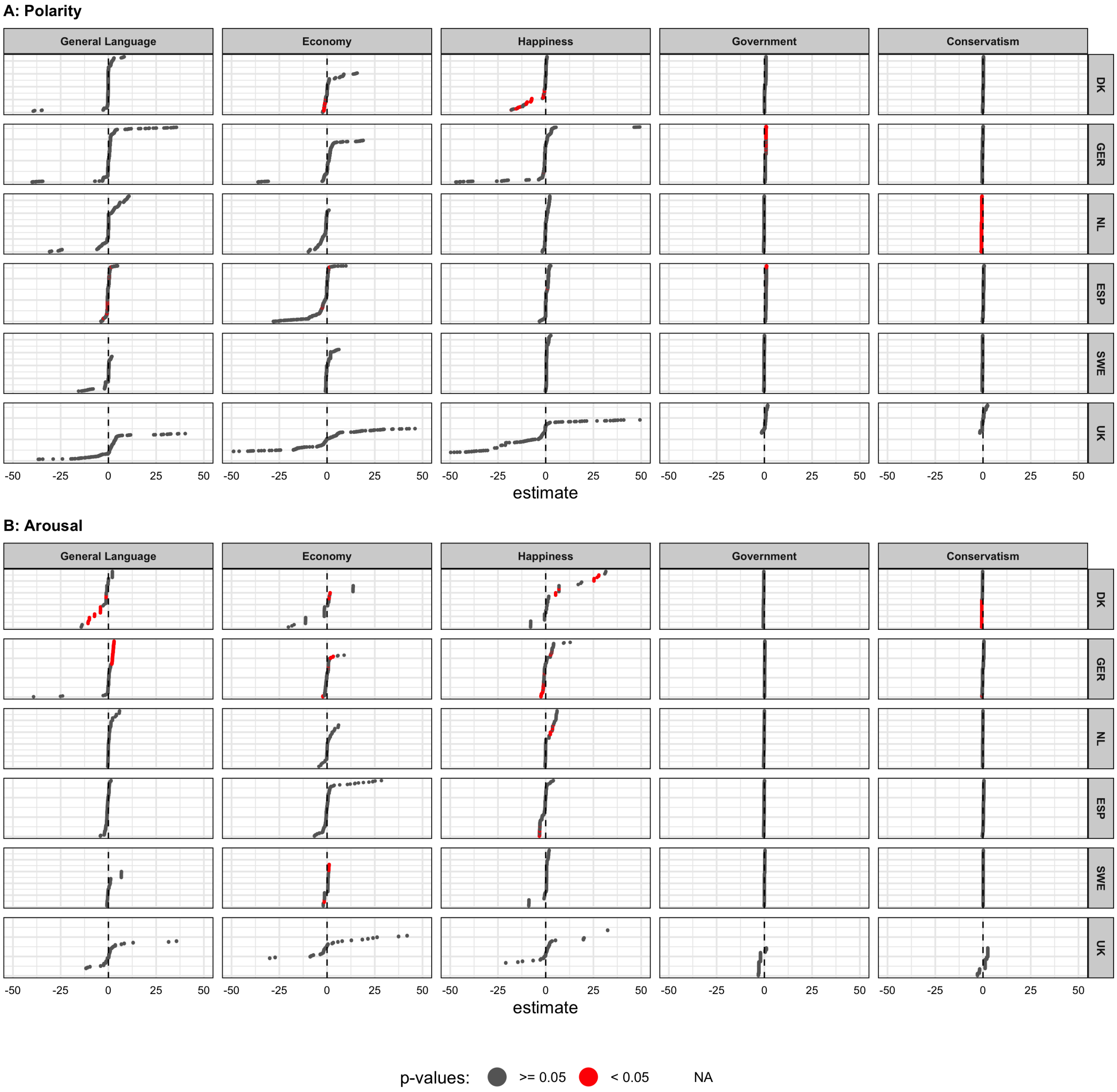

Note: Results from the multiverse of multilevel models explaining polarity and arousal in party manifestos. Data from CMP (3). Country and time period selection equivalent to main analyses using parliamentary data. Effects with $p < .05$ are shown in red, effects with $p >= .05$ are shown in black. The dashed line indicates an effect of 0. All continuous variables have been standardized.

B.2 Replication with leader speeches

Figure B2: Specification curves of tone in EU leader speeches across multiverse of models

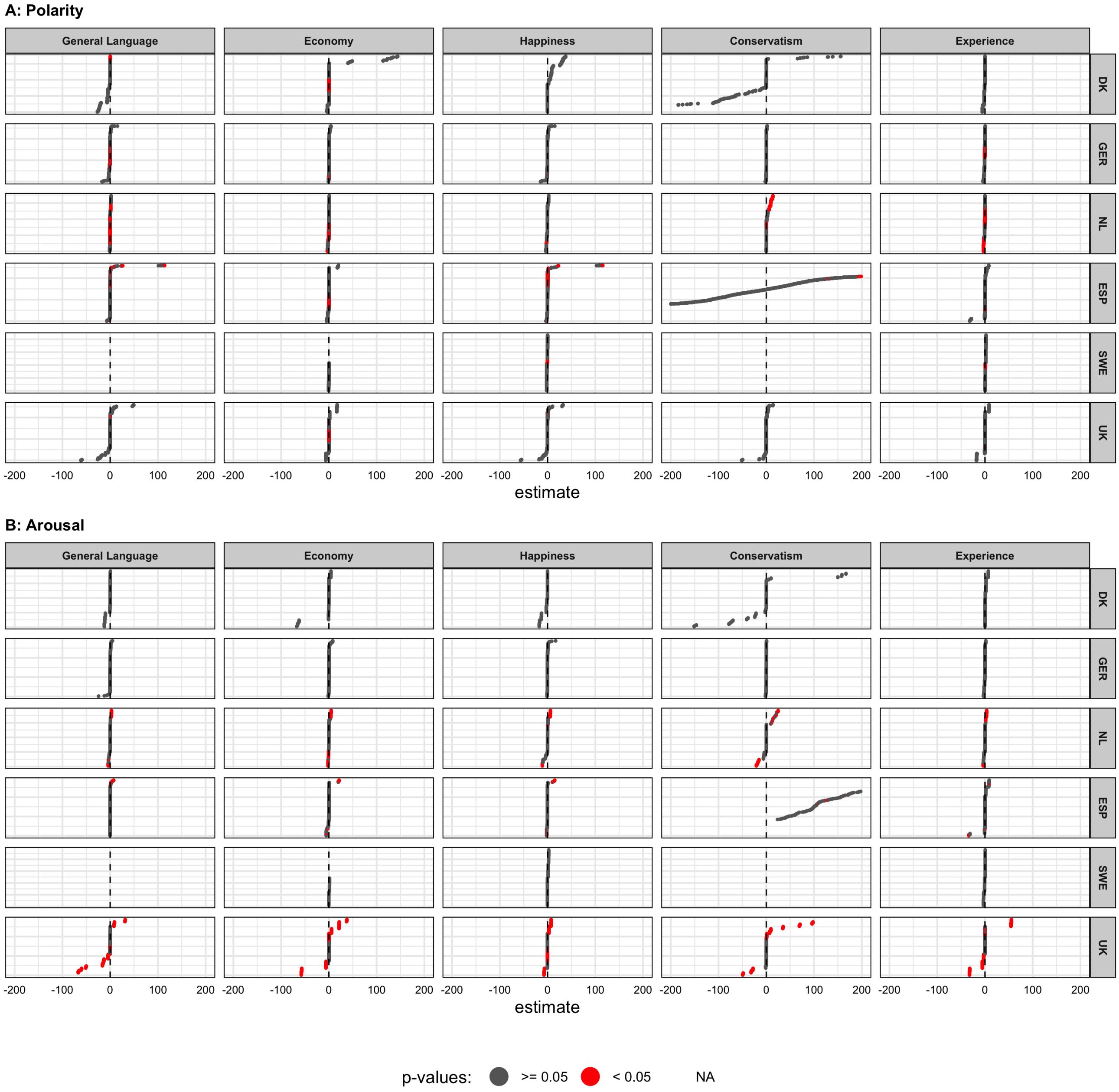

Note: Results from the multiverse of multilevel models explaining polarity and arousal in the speeches of EU leaders. Data from EUSpeech v2 dataset (4). Time periods covered in the dataset vary between countries. Country and time period selection limited to main analyses using parliamentary data. No estimates for general language and conservatism in Sweden possible due to very short time period and lack of variation in data on these variables. No leader speech data available for Austria. Effects with $p < .05$ are shown in red, effects with $p >= .05$ are shown in black. The dashed line indicates an effect of 0. All continuous variables have been standardized.

C Validation of the Joint Sentiment-Topic Model on Parliamentary Speeches

Measuring tone in parliamentary speeches using JST models generally improve the accuracy of the automated emotion measures compared to the more standard approach of just using sentiment dictionaries and have been extensively validated (5; 6; 7). We here present a multi-language validation of JST with hand-coded speeches from the British House of Commons, the German Bundestag, and the Dutch Tweede Kamer. From the ParlSpeech v2 dataset (8), we sampled 200 speeches from each of these three parliaments, excluding speeches from the chair because those mainly concern parliamentary procedures and announce speaker turns. We recruited 9 native speakers in total (3 per parliament/language) whom we asked to judge if a speech was broadly positive, neutral, or broadly negative. Each speech was coded by 3 coders. Since we were interested in the overall tone of the speech we instructed coders to not focus on particular aspects of the speech, but to rate their overall impression of its polarity. For this reason we introduced an upper limit of 500 words per speech because for longer speeches, often covering multiple topics, this coding task would have been too hard. We also excluded speeches with fewer than 50 words because they carry little substantive content. Did we expect agreement between coders? High agreement between coders is important when coding policy dimensions or topics. Regarding polarity we expect coders to disagree to some extent, because differences between coders reflect the ambiguity of language regarding polarity categories (9; 10).

We assigned numeric values to each coding (-1, 0, +1) and took the mean of the three coder ratings for each speech to arrive at a finer-grained continuous polarity score. In all three corpora, coders originally judged a majority of speeches as broadly negative (UK: 48%, DE: 50%, NL: 50%) or neutral (UK: 37%, DE: 34%, NL: 28%), with only a much smaller proportion of speeches codedas broadly positive (UK: 15%, DE: 16%, NL: 22%). Such skewed distributions provide a challenge for machine learning algorithms, especially compared to curated and balanced training sets like movie reviews. Parliamentary speeches thus present a challenging test for the JST model.

To get JST estimates for these 200 speeches, we ran JST models with 10,000 speeches from each parliament (using the same criteria with which the 200 coded speeches were initially selected). Since JST is semi-supervised, it requires a sentiment dictionary as a supervised input. We use the multilingual Lexicoder Sentiment dictionary (LSD) (11), originally developed for political news coverage (12), as a partly-context-specific dictionary. This dictionary has been validated on parliamentary speeches for all EU languages (11). When estimated, JST returns three separate probabilities for the polarity labels neutral, positive, and negative. To arrive at a continuous polarity measure per speech (similar to the dictionary measures) we subtracted the probability for the negative label from the probability for the positive label. Thus, a speech with a JST estimate of 0.11 (Neutral), 0.65 (Positive), 0.24 (Negative) received an overall polarity score of 0.41. To see if our JST models outperformed the (already validated) plain JST dictionary applications, we also calculated polarity measures using each dictionary alone. To this end we counted the share of words identified by each dictionary category (positive and negative) in each speech, and subtracted the share of negative matches from the share of positive matches. Since choosing an optimal number of topics a priori is difficult, we varied k between 5 and 30 in increments of 5 and estimated a JST model each time. Finally, we estimated each model ten times and averaged their results.

How do JST and dictionary polarity scores perform relative to each other? Table C1 presents the (Pearson) correlations between (1) Lexicoder sentiment dictionaries and human-coded polarity and (2) JST estimates and human-coded polarity. In all cases, the polarity estimates obtained by JST have a higher overlap with human-coded polarity than dictionary scores alone. This difference

is especially pronounced in the Dutch case, where the off-the-shelf dictionary application performs worst. Our findings of dictionary performance are in line with previous work showing low performance when using general domain sentiment dictionaries within a specific domain like politics (13; 14). We found that the overlap between human and machine polarity measures improves as we aggregate the level of analysis. This is important, because in our analyses we use highly aggregated data at the speaker-quarter level. In our validation across the three coded datasets, we use a small subset and selected parties with over 10 speeches and computed automated scores based on their aggregated texts. While do no have enough coded data to this at scale across all parties, the results are in line with previous work on dictionary methods showing significantly increased accuracy when using larger units of texts (see the work by Proksch and colleagues (11) who show this for party-aggregates of EU legislative speeches, demonstrating correlations between 0.7 and 0.9 with human coding depending on the language). Overall, the JST method consistently surpassed traditional dictionary applications.

Corpus	level	% r Dictionary-Human Coding	% r JST-Human Coding
UK House of Commons	speech	0.46	**0.52**
DE Bundestag	speech	0.40	**0.45**
NL Tweede Kamer	speech	0.26	**0.36**

Table C1: Mean Pearson correlations between automated measures and human coding, dictionary application vs. JST. Note: JST results are averaged over multiple JST models with varying topic number k from 5 to 30 in steps of 5 and 10 runs per model.

D Preanalysis plan

D.1 Deviation from preanalysis plan

We preregistered our analytical strategy before compiling or analyzing the data on OSF (https://osf.io/ur5xg/). We further preregistered a follow-up study using additional data sources after the results were known. We summarize our deviations from these plans in table D1.

Preregistration element	Deviation	Reason
Hypothesis wording	changed *well-being* to *economic performance*	Better distinction from subjective happiness
Hypothesis wording	changed *general langauge use* to *general langauge use in society*	Make clear that we refer to how languge is generally used in the society
Order of hypotheses	H3 (subjective happiness) → H4<br>H4 (economy) → H3<br>H5 (experience) → H6<br>H6 (ideology) → H5	More coherence in ordering the hypotheses, going from macro trends to individual differences
Case selection	Dropped speeches from Czech Republic	No language stemmer readily available in R
IV: General language	Dropped option using newspaper data	No data source available
Control variable: Legislator replacement	Dropped option calculating replacement based on all MPs in parliament	Not sensible how this relates to speech differences
Appendix: Leader and manifesto analysis	OLS models instead of multilevel models	not enough groups to estimate ML models

Table D1: Deviations from preanalysis plan

References

[1] Luca Bernardi, Daniel Bischof, and Ruud Wouters. The public, the protester, and the bill: Do legislative agendas respond to public opinion signals? *Journal of European Public Policy*, 0(0):1–22, 2020.

[2] Suzanna De Boef and Luke Keele. Taking time seriously. *American Journal of Political Science*, 52(1):184–200, 2008.

[3] Andrea Volkens, Werner Krause, Pola Lehmann, Theres Matthieß, Nicolas Merz, Sven Regel, and Bernhard Weßels. The Manifesto Data Collection. Manifesto Project (MRG/CMP/MARPOR). 2019.

[4] Gijs Schumacher, Nicolai Berk, Christian Pipal, Jaroslaw Kantorowicz, Martijn Schoonvelde, Denise Traber, and Erik de Vries. EUSpeech V2. 2020.

[5] Chenghua Lin and Yulan He. Joint sentiment/topic model for sentiment analysis. In *International Conference on Information and Knowledge Management, Proceedings*, pages 375–384, 2009.

[6] Chenghua Lin, Yulan He, Richard Everson, and Stefan Rüger. Weakly supervised joint sentiment-topic detection from text. *IEEE Transactions on Knowledge and Data Engineering*, 24(6):1134–1145, 2012.

[7] Christian Pipal, Martijn Schoonvelde, and Gijs Schumacher. Taking Context Seriously: Joint Estimation of Sentiment and Topics in Textual Data. Publisher: OSF Preprint, 2021.

[8] Christian Rauh and Jan Schwalbach. *The ParlSpeech V2 Data Set: Full-Text Corpora of 6.3 Million Parliamentary Speeches in the Key Legislative Chambers of Nine Representative Democracies.* Harvard Dataverse, v2 edition, 2020.

[9] Alina Andreevskaia and Sabine Bergler. Mining WordNet for fuzzy sentiment: Sentiment tag extraction from WordNet glosses. In *EACL 2006 - 11th Conference of the European Chapter of the Association for Computational Linguistics, Proceedings of the Conference*, pages 209–216, 2006.

[10] Pero Subasic and Alison Huettner. Affect analysis of text using fuzzy semantic typing. *IEEE Transactions on Fuzzy Systems*, 9(4):483–496, 2001.

[11] Sven Oliver Proksch, Will Lowe, Jens Wäckerle, and Stuart Soroka. Multilingual Sentiment Analysis: A New Approach to Measuring Conflict in Legislative Speeches. *Legislative Studies Quarterly*, 44(1):97–131, February 2019.

[12] Lori Young and Stuart Soroka. Affective News: The Automated Coding of Sentiment in Political Texts. *Political Communication*, 29(2):205–231, 2012.

[13] Wouter van Atteveldt, Mariken A. C. G. van der Velden, and Mark Boukes. The Validity of Sentiment Analysis: Comparing Manual Annotation, Crowd-Coding, Dictionary Approaches, and Machine Learning Algorithms. *Communication Methods and Measures*, 15(2):121–140, April 2021.

[14] Tobias Widmann and Maximilian Wich. Creating and Comparing Dictionary, Word Embedding, and Transformer-Based Models to Measure Discrete Emotions in German Political Text. *Political Analysis*, pages 1–16, June 2022. Publisher: Cambridge University Press.
